# Chicken Peripheral Blood Mononuclear Cells Response to Avian Leukosis Virus Subgroup J Infection Assessed by Single-Cell RNA Sequencing

**DOI:** 10.3389/fmicb.2022.800618

**Published:** 2022-03-14

**Authors:** Xiaoyun Qu, Xiaobo Li, Ziwei Li, Ming Liao, Manman Dai

**Affiliations:** ^1^National and Regional Joint Engineering Laboratory for Medicament of Zoonosis Prevention and Control, Guangdong Provincial Key Laboratory of Zoonosis Prevention and Control, College of Veterinary Medicine, South China Agricultural University, Guangzhou, China; ^2^Core Facilities for Medical Science, Sun Yat-sen University, Guangzhou, China

**Keywords:** scRNA-seq, chicken, PBMCs, ALV-J, T cell

## Abstract

Chicken peripheral blood mononuclear cells (PBMCs) exhibit wide-ranging cell types, but current understanding of their subclasses, immune cell classification, and function is limited and incomplete. Here we performed single-cell RNA sequencing (scRNA-seq) of PBMCs in Avian leukosis virus subgroup J (ALV-J) infected and control chickens at 21 days post infection (DPI) to determine chicken PBMCs subsets and their specific molecular and cellular characteristics. Eight cell populations and their potential marker genes were identified in PBMCs. T cell populations had the strongest response to (ALV-J) infection, based on the detection of the largest number of differentially expressed genes (DEGs), and could be further grouped into four subsets: activated CD4^+^ T cells, Th1-like cells, Th2-like cells, and cytotoxic CD8^+^ T cells. Furthermore, pseudotime analysis results suggested that chicken CD4^+^ T cells could potentially differentiate into Th1-like and Th2-like cells. Moreover, ALV-J infection activated CD4^+^ T cell was probably inclined to differentiate into Th1-like cells. Compared to the control PBMCs, ALV-J infection also had an obvious impact on PBMCs composition. B cells showed inconspicuous response and their numbers decreased in PBMCs from ALV-J infected chicken. Proportions of cytotoxic Th1-like cells and CD8^+^ T cells increased in the T cell population of PBMCs from ALV-J infected chicken, which were potentially key mitigating effectors against ALV-J infection. More importantly, our results provide a rich resource of gene expression profiles of chicken PBMCs subsets for a systems-level understanding of their function in homeostatic condition as well as in response to viral infection.

## Introduction

Adaptive immunity is known to play a vital protective role against avian viral infections. However, many interesting scientific questions about avian T cell or B cell immunity remain unresolved (Dai et al., 2019). For example, many important marker genes of the chicken immune cells are unknown, including effector or memory T cells and B cells. This greatly limit the immune cell phenotyping and subsequent immune function and mechanistic studies. Specifically, effector CD4^+^ T cells can differentiate into many T helper (Th) subsets, resulting in the production of different cytokines and effector functions. The Th1-Th2 paradigm is reported to exist in chickens (Degen et al., 2005). However, whether this paradigm holds true at the cellular and molecular levels and whether chicken Th cells can become terminally polarized to a Th1 or Th2 phenotype remain to be verified. Furthermore, it is also important and interesting to thoroughly characterize the molecular signatures of the CD4^+^ T, CD8^+^ T cells, and B cells during homeostasis and after pathogen exposure.

Chicken peripheral blood mononuclear cells (PBMCs), which contain various cells including T cells, B cells, natural killer (NK) cells, monocytes, and dendritic cells (DCs), are reported to execute important functions in eliminating avian viral infections (Bi et al., 2018; Dai et al., 2020a; Dai et al., 2021). However, such studies are usually based on bulk PBMCs measurements overlooking the complexity of diverse cell types. Recent advances in single-cell RNA-seq (scRNA-seq) allow the breakdown of complex tissues or host compartments into individual cell types for exploring their relevance in health and disease (Hen-Avivi and Avraham, 2018). scRNA-seq has already been used to investigate the immune response of human peripheral blood cells under infection of pathogenic microorganisms including salmonella, severe acute respiratory syndrome corona virus 2 (SARS-CoV-2), and influenza virus (Ben-Moshe et al., 2019; Wilk et al., 2020; Zhu et al., 2020). In chickens, the cell lineage characteristics in some tissues have been identified using scRNA-seq, such as the developing chicken limb (Feregrino et al., 2019), chicken skeletal muscle (Li et al., 2020), and embryonic chicken gonad (Estermann et al., 2020). However, no reported study used scRNA-seq to determine chicken immune cell subsets or lineages. Moreover, to our knowledge, scRNA-seq technology has not even been applied to study chicken PBMCs responses to any viral infection.

Avian leukosis virus subgroup J (ALV-J), an avian oncogenic retrovirus, causes enormous economic losses in the global poultry industry as there are currently no vaccines or drug treatments (Feng and Zhang, 2016). A potential vaccine for ALV-J has been reported to induce significantly increased CD4^+^ and CD8^+^ T cell proportion as well as IL-4 and IFN-γ levels in immunized chickens (Xu et al., 2015). Unfortunately, few studies explored the specific T cell phenotype or function against ALV. A full understanding of the ALV-specific cellular immune response in chickens is likely the premise for developing effective vaccines. In our previous study, we found that ALV-J viremia was eliminated by 21 days post infection (DPI) when a significantly up-regulated CD8^+^T cell proportion and a very low serum antibody level in the peripheral blood were detected (Dai et al., 2020a). As described above, PBMCs contains many cell types besides CD8^+^ T cells and B cells. Usually, these immune cells are able to form a complex network of communications that maintains an orchestrated and dynamic immune response to eliminate invading pathogens (Ben-Moshe et al., 2019). Hence, elucidating different chicken PBMCs subsets responding to viral infection is very important.

In the current study, we performed 10x scRNAseq on PBMCs from ALV-J infected and uninfected chickens at 21 DPI to comprehensively identify PBMCs subsets and characterize their specific cellular and molecular responses after viral infection. More importantly, we provide evidence to show that chicken Th cells can be terminally polarized to a Th1 or Th2 phenotype. Moreover, we have developed an extensive catalog of candidate marker genes and immune factors for identifying known and unknown chicken immune cells and their functions.

## Materials and Methods

### Ethics Statement

All animal research projects were sanctioned by the South China Agriculture University Institutional Animal Care and Use Committee (identification code: 2019076, 10 June, 2019). All animal procedures were performed according to the regulations and guidelines established by this committee and international standards for animal welfare.

### Sample Preparation

Peripheral blood mononuclear cells samples from ALV-J infected and control chickens at 21 DPI were prepared as previously described (Dai et al., 2020a). Briefly, 4-week-old specific-pathogen-free (SPF) chickens were inoculated intraperitoneally at a dose of 0.8 mL (10^4^ TCID_50_/0.1 mL) of ALV-J strain CHN06, and the virus was eliminated at 21 DPI when a significantly up-regulated CD8^+^ T cell ratio in PBMCs was detected compared to the control group injected with 0.8 mL PBS alone (Dai et al., 2020a). To further explore the immune signature of various lymphocytes in PBMCs, we further investigated the single-cell survey of the chicken PBMCs response to ALV-J infection at 21 DPI.

### Single Cell Suspension for 10x scRNAseq

Pooled PBMCs from blood of three ALV-J infected chickens or three control chickens at 21 DPI were, respectively, resuspended in PBS (calcium and magnesium-free; Gibco, Thermo Fisher Scientific, Waltham, MA, United States) with 0.4% bovine serum albumin (BSA; Solarbio, China), followed by passing through a 40 μm cell strainer (Biosharp, China). Cell concentration and viability were assessed using Trypan Blue and a Neubauer hemocytometer (Sigma-Aldrich, St. Louis, MO, United States). Cell viability in both samples was about 80%. Subsequently, the cell density was adjusted to 1 × 10^6^ cells/mL. High quality single cell suspensions were subjected to encapsulation using a 10x Genomics v.3 kit (10x Genomics, United States).

### Library Preparation for 10x scRNAseq

Single cell encapsulation, complementary DNA (cDNA) library synthesis, RNA-sequencing, and data analysis were completed by Gene *Denovo* (Guangzhou, China). The single-cell suspensions were bar-coded and reverse-transcribed into scRNA-seq libraries using the Chromium Single Cell 3′ Gel Bead-in Emulsion (GEM) Library and Gel Bead Kit (10x Genomics) according to the manufacturer’s protocol. Briefly, single cells of the ALV-J infected and control PBMCs at 21 DPI were separately barcode-labeled and mixed with reverse transcriptase into GEMs; the cDNA library was then using PCR with the sequencing primers R1 and R2, and subsequently ligated to Illumina sequencing adapters with P5 and P7. Finally, the cDNA libraries were sequenced on the Illumina 10x Genomics Chromium platform (Illumina Novaseq 6000). An average of 18,895 reads per cell in the ALV-J infected PBMCs and 30,540 mean reads per cell in the control PBMCs were obtained, respectively.

### Single-Cell RNA Sequencing Data Processing and Analysis

#### Data Processing

Cell Ranger^1^ (v3.1.0) uses an aligner called STAR,^2^ which performs splicing-aware alignment of reads to the chicken genome of GRCg6a (Zerbino et al., 2018). Only reads that are confidently mapped to the transcriptome are used for Unique Molecular Identifier (UMI) counting. Cells with unusually high numbers of UMIs (≥8,000) or mitochondrial gene percentage (≥10%) were filtered out. Cells with <500 or >4,000 gene counts were also excluded. Using the R package Seurat v.2.3.2 (Butler et al., 2018), UMI counts were then Log-normalized and any variation due to the library size or mitochondrial UMI count proportion was then regressed via a variance correction using the function ScaleData.

#### Dimensionality Reduction and Visualization

Top 50 significant principal components (PCs) were determined for downstream clustering and dimensional reduction following the jackStraw procedure (Butler et al., 2018). Then, Seurat was used to implement the graph-based clustering approach (resolution setting as 0.6) (Levine et al., 2015; Xu and Su, 2015). T-distributed Stochastic Neighbor Embedding (t-SNE) (Linderman et al., 2019) or Uniform Manifold Approximation and Projection (UMAP) (Becht et al., 2019) in Seurat were used to visualize and explore these datasets.

#### Differentially Expressed Gene (Up-Regulation) Analysis per Cluster

We used the Wilcoxon rank sum test (Camp et al., 2017) to identify differential expression for a single cluster, compared to all other cells. We identified up-regulated DEGs according to the following criteria: (1) *P* value ≤ 0.01; (2) logFC ≥ 0.360674 (logFC means log fold-change of the average expression between the two compared groups); (3) The proportion of cells in which the gene is detected in a specific cluster >25%. Gene ontology (GO) enrichment analysis selects all GO terms that are significantly enriched in DEGs compared to the genome background; furthermore, the DEGs are filtered to correspond to biological functions. All DEGs were mapped to GO terms in the Gene Ontology database (The Gene Ontology Consortium [GOC], 2019), gene numbers were calculated for every term, and significantly enriched GO terms in DEGs compared to the genome background were identified by hypergeometric testing to identify the main features of each cluster. Finally, Pearson’s correlation analysis was performed to investigate correlations between different clusters based on the levels of gene expression.

#### Marker Gene Analysis

We further selected the top five expressed genes in each cluster as marker genes according to the result of differentially expressed genes (parameters used: logFC > 0.25; min_pct > 0.25; *p* value < 0.01). The expression distribution of each marker gene was then demonstrated using bubble diagrams. We also checked the expression of classical marker genes of chicken immune cells, e.g., CD4 T cell (*CD3D*, *IL7R*, and *CD4*), B cell (BCL11A, Bu-1, and BLB2), CD8 T cell (*CD3D*, *CD8A*, and *GNLY*), Dendritic Cell (DC, *CD80*, *CD86*, and *BLB2*), and Natural killer cell (NK, *CD8A*, *CD5*, and *CD44*) (see Additional file 2 in Supplementary Material, Figures 2, 4) (Stewart et al., 2013).

**FIGURE 1:**
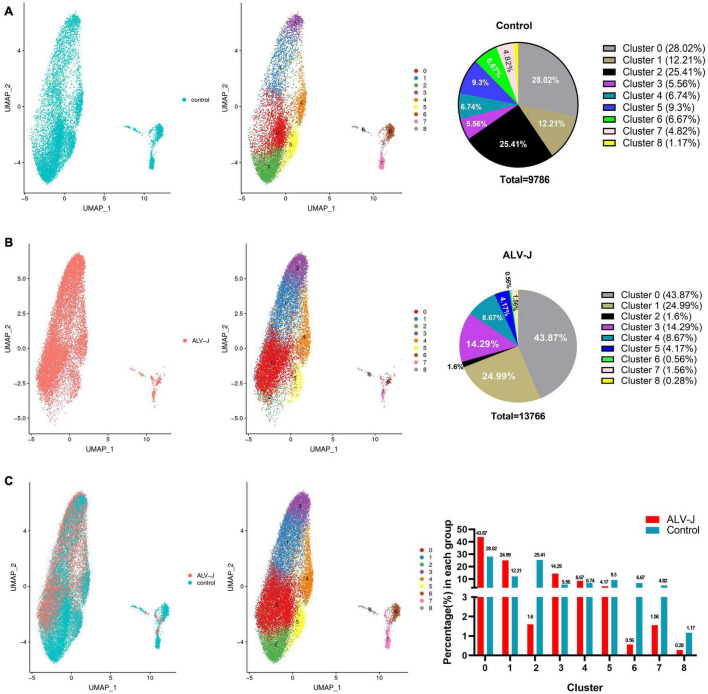
Single-cell profiling of cell populations in chicken peripheral blood mononuclear cells (PBMCs) collected from avian leukosis virus subgroup-J (ALV-J)-infected and control chickens at 21 days post infection (DPI). **(A)** Uniform Manifold Approximation and Projection (UMAP) display of all cell populations and their proportions in the control PBMCs. **(B)** UMAP display of all cell populations and their proportions in PBMCs from ALV-J infected chicken. **(C)** Cell distribution and proportion of each cluster in PBMCs from ALV-J infected and control chickens.

**FIGURE 2:**
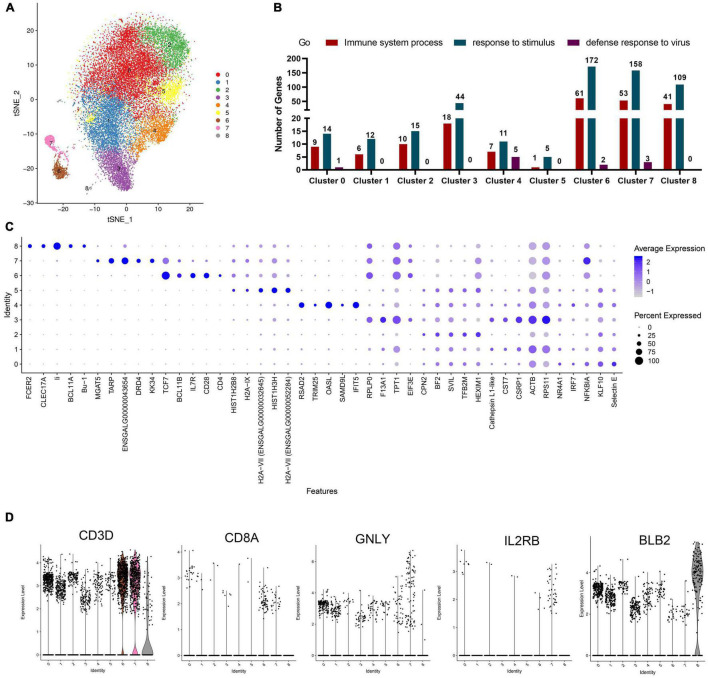
Analysis of cell types of each cluster in chicken PBMCs. **(A)** t-Distributed Stochastic Neighbor Embedding (t-SNE) projection representing the eight clusters of cells identified in the chicken PBMCs (unified set of control and ALV-J infection samples). **(B)** The statistics of genes involved in the GO terms “immune system process,” “response to stimulus,” and “defense response to virus,” as analyzed in each cluster. **(C)** Top five DEGs (x-axis) identified in each cluster (y-axis). Dot size represents the proportion of cells in the cluster that express the gene; intensity indicates the mean expression level (*Z*-score) in the cells, relative to those from other clusters. **(D)** Expression levels of the characteristic marker genes (*CD3D*, *CD8A*, *GNLY*, *IL2RB*, and *BLB2*) in PBMCs clusters.

**FIGURE 3:**
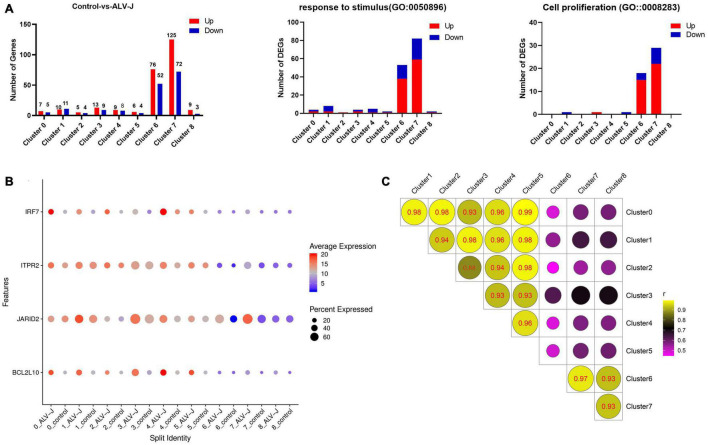
Differentially expressed gene (DEG) analysis in PBMCs from ALV-J infected and control chickens within cell populations**. (A)** Bar graphs showing all up-regulated (red) and down-regulated (blue) DEGs, and the number of DEGs involved in the GO terms “response to stimulus” and “cell proliferation” in PBMCs from ALV-J infected chicken compared to control PBMCs within each cluster. **(B)** Dot plot representing selected DEGs (*IRF7*, *ITPR2*, *JARID2*, and *BCL2L10*) expressed in eight clusters within which cells from the ALV-J infected sample were compared with the control sample. The intensity represents the expression level, while dot size represents the proportion of cells expressing each gene. **(C)** Pearson’s Correlation analysis of different cell populations based on gene expression levels.

**FIGURE 4:**
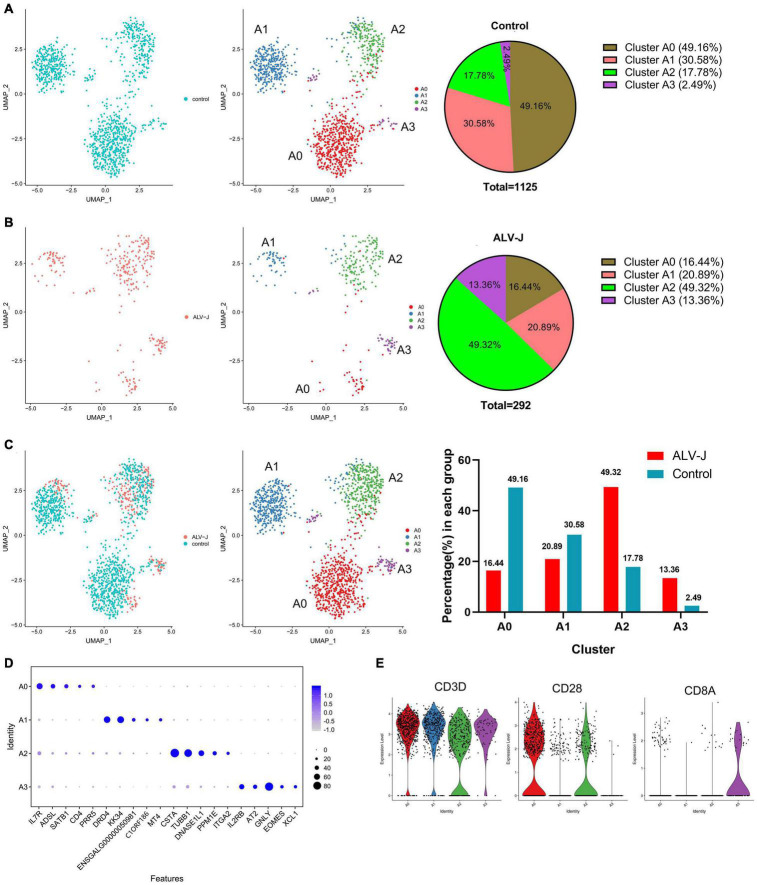
T cell population of clusters 6 and 7 were further divided into four distinct cell populations**. (A)** UMAP display of the cell populations and their proportions of the T cell sub clustering (clusters 6 and 7) in the control PBMCs. **(B)** UMAP display of the cell populations and their proportions of the T cell sub clustering (clusters 6 and 7) in PBMCs from ALV-J infected chicken. **(C)** The regrouped T cell distribution and proportion of each cluster in PBMCs from ALV-J infected and control chickens. **(D)** Top five DEGs (x-axis) identified in clusters A0–A3 (y-axis). **(E)** Expression levels of characteristic marker genes (*CD3D*, *CD28*, and *CD8A*) in clusters A0–A3.

### Pseudo Temporal Ordering of Cells

Single cell trajectory was analyzed using a matrix of cells and gene expressions in Monocle 2 (v.2.6.4) (Trapnell et al., 2014). Monocle reduces the space in which cells are embedded to two dimensions and orders the cells (parameters used: sigma = 0.001, lambda = NULL, param.gamma = 10, tol = 0.001). Once the cells were ordered, the trajectory (with a tree-like structure, including tips and branches) could be visualized in the reduced dimensional space.

### Differentially Expressed Gene Analysis in Cell Populations of the Peripheral Blood Mononuclear Cells From the Avian Leukosis Virus Subgroup J Infected and Control Chickens

To explore the response of each cluster in PBMCs, we further analyzed the DEGs in cell populations of PBMCs from the infected and control chickens using Seurat’s R package. A hurdle model in MAST (Model-based Analysis of Single-cell Transcriptomics) (Finak et al., 2015) was used to identify DEG group in one cluster. DEGs between the ALV-J infected and control samples were identified by the following criteria: (1) | log2FC| ≥ 1; (2) *P* value ≤ 0.01; and (3) proportion of cells in which the gene was detected in a specific cluster >25%. Identified DEGs were subsequently subjected to GO enrichment analysis as described above.

### Protein-Protein Interaction Network Analysis

The interaction network of the candidate DEGs was constructed using String v.10.0 and Cytoscape (v.3.3.0) software. Specifically, the protein-protein interaction network was identified using String (Szklarczyk et al., 2015), which determined genes as nodes and interactions as lines in a network. The final network file was visualized using Cytoscape software (Shannon et al., 2003) to present a core and hub gene biological interaction.

## Results

### Single-Cell Transcriptomics Identified Eight Distinct Cell Populations in the Peripheral Blood Mononuclear Cells Collected From Avian Leukosis Virus Subgroup J-Infected and Control Chickens at 21 Days Post Infection

We used the 10x Genomics platforms to perform 3′ scRNA-seq on PBMCs collected from ALV-J infected and PBS-treated control chickens at 21 DPI, respectively. Details on the statistics of scRNA-seq are summarized in Additional file 1 in Supplementary Material. A total of 13, 766 cells in the PBMCs from ALV-J infected chicken and 9,786 cells in the control PBMCs were profiled, and eight distinct clusters were obtained and visualized using UMAP (Figure 1) or t-SNE (Figure 2A). Clusters 6, 7, and 8 occupied a very small proportion in chicken PBMCs (Figure 1C), and the proportion of clusters 0, 1, and 3 were increased in the PBMCs from ALV-J infected chicken when compared to the control. Conversely, the proportion of clusters 2, 6, 7, and 8 were distinctly reduced in the PBMCs from ALV-J infected chicken at 21 DPI (Figures 1A–C).

For further analyzing the immune signatures of each cluster in chicken PBMCs, we identified the up-regulated DEGs in a single cluster compared to all other cells, and analyzed the DEGs enriched in the GO terms “immune system process,” “response to stimulus,” and “defense response to virus.” The results showed that most immune-related DEGs (DEGs enriched in the above three Go terms) were mainly detected in clusters 6, 7, and 8 (Figure 2B, Additional files 2, 3 in Supplementary Material), which implied that, in PBMCs, these clusters are the main effectors responded to pathogenic stimuli. Additionally, the expression levels and the proportion of cells expressing the top five genes in each cluster are shown in a dot plot (Figure 2C, Additional files 4, 5 in Supplementary Material); these need to be confirmed in future research and are proposed to be used as marker genes for each cluster of chicken PBMCs.

Based on the expression of classical CD3 marker, we could define clusters 6 and 7 as T cells (Figure 2D). Cluster 6 mainly included CD4^+^ T cells (CD3^+^CD4^+^IL7R^+^CD28^+^) (Figures 2C,D). The marker gene, *KK34* in Cluster 7 is reported to encode an IL-5-like transcript that was specifically expressed by avian γδT cells in the peripheral blood, which may mediate T helper 2 (Th2)-cytokine-dependent allergy (Koskela et al., 2004). Meanwhile, other studies reported that dopamine receptor (*DRD4*) is involved in Th2 cell differentiation and inflammation (Wang W. et al., 2019). Therefore, we defined cluster 7 as Th2-like cell (CD3^+^KK34^+^DRD4^+^; Figures 2C,D). In addition, we found that a few cytotoxic CD8^+^ T cells (CD3^+^CD8^+^GNLY^+^) mixed in clusters 6 and 7 (Figure 2D). Therefore, we planned to subdivide clusters 6 and 7 for a more detailed display of data in the following analysis. Additionally, cluster 8 should be B cells according to the expression of the known marker genes, BCL11A, Bu-1 (ENSGALG00000015461), and Class II (also named as BLB2) (Figures 2C,D). Interestingly, the top five genes in cluster 4 were mainly interferon stimulating genes (ISGs), including *RSAD2*, *TRIM25*, *OASL*, and *IFIT5*, which implied that cluster 4 also contained a type of important antiviral immune cell (Figure 2C). Therefore, we defined cluster 4 as ISG expressing cells in PBMCs, which needs to be further verified in future studies. Unfortunately, we were unable to define clusters 0, 1, 2, 3, and 5 based on the limited known chicken cell-type markers and the top five genes expressed by cells in these clusters.

### Most Differentially Expressed Genes Were Detected in the T Cell Population (Clusters 6 and 7) in Response to Avian Leukosis Virus Subgroup J Infection at 21 Days Post Infection

We calculated the DEGs between cell populations of PBMCs from the ALV-J infected and control chickens at 21 DPI using Seurat. It was found that the total number of DEGs and the DEGs enriched in the GO terms “response to stimulus” and “cell proliferation” were largely detected in clusters 6 and 7 (Figure 3A, Additional file 6 in Supplementary Material), indicating that cells in clusters 6 and 7 might have played an important role during the antiviral response to ALV-J in the infected chicken. We further discovered that the decreased proportion of clusters 6 and 7 in PBMCs from ALV-J infected chicken might be a result of the up-regulated pro-apoptotic factors, *ITPR2* (van Es et al., 2007) and *JARID2* (Gennart et al., 2015) (Figures 1C, 3B, Additional file 6 in Supplementary Material). Next, an immune related DEG analysis was performed after sub clustering of clusters 6 and 7 in the study outlined below.

Meanwhile, some DEGs in other clusters were analyzed. We found that an important immune gene, interferon regulator 7 (*IRF7*), revealed up-regulation in clusters 0, 1, 2, and 3 in response to ALV-J infection at 21 DPI (Figure 3B, Additional file 6 in Supplementary Material). We also found that the anti-apoptotic gene *BCL2L10* (Zang et al., 2015) was up-regulated in clusters 0, 1, 3, 4, and 5 of PBMCs from ALV-J infected chicken (Figure 3B, Additional file 6 in Supplementary Material). Besides, Pearson’s correlation analysis indicated that clusters 0, 1, 2, 3, 4, and 5 were strongly correlated between each other, but displayed very low correlation with clusters 6, 7, and 8 (corresponding to T cells and B cells; Figure 3C). And at 21 DPI, the late infection stage, we detected most DEGs in the T cell population (clusters 6 and 7) (Figure 3A, Additional file 6 in Supplementary Material). So, the function of cells in clusters 0, 1, 2, 3, 4, and 5 may be worked at other infection stage such as the early infection stage.

### T Cell Populations of Clusters 6 and 7 Could Be Further Divided Into Four Distinct Cell Populations in the Peripheral Blood Mononuclear Cells

The above-mentioned T cell populations of clusters 6 and 7, both in PBMCs from the ALV-J infected or control chicken, were further divided into four distinct clusters (named as clusters A0–A3) and visualized using UMAP (Figures 4A–C). Compared to those in the control PBMCs, the proportions of clusters A0 and A1 in the PBMCs from ALV-J infected chicken decreased. Conversely, the proportions of clusters A2 and A3 significantly increased in PBMCs from ALV-J infected chicken (Figure 4C). Next, the top five genes expressed in clusters A0–A3 were picked as potential marker genes and they are shown in a dot plot (Figure 4D, Additional files 7, 8 in Supplementary Material). Based on the classical marker genes and our above analysis, we considered cluster A0 as CD4^+^ T cells (CD3^+^CD4^+^IL7R^+^CD28^+^), cluster A3 as cytotoxic CD8^+^ T cells (CD3^+^CD8^+^IL2RB^+^GNLY^+^), and cluster A1 as Th2 like cells (CD3^+^KK34^+^DRD4^+^) (Figures 4D,E). Besides, cluster A0 contained some CD4^+^ CD8^+^T cells based on the low expression level of CD8A (Figure 4E), which could not be separated with CD4^+^ T cells.

**FIGURE 5:**
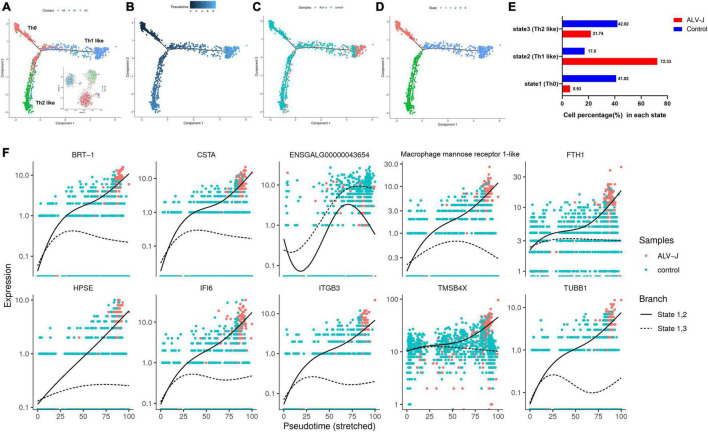
Pseudotime analysis of clusters A0, A1, and A2**. (A)** Mapping of clusters A0 (Th0), A1 (Th2-like), and A2 (Thl-like) to the pseudotime trajectory. **(B)** Pseudotime trajectory calculated from all cells of clusters A0–A2 in the control and ALV-J infected samples. Darker colored dots represent a shorter pseudotime and earlier differentiation period. **(C)** Mapping of cells in control and ALV-J infected samples to the pseudotime trajectory. **(D)** The cell states of pseudotime trajectory partitioned from all cells of clusters A0–A2 in the control and ALV-J infected samples. **(E)** Cell proportion of each state between the ALV-J infected and control samples. **(F)** Dynamics of the top 10 branching DEGs. Full line: state 1, 2; imaginary line: state 1, 3.

Although we were unable to define cluster A2 based on their top five genes and a few known markers of various chicken cell-types, using pseudotime analysis, we believe that clusters A0 (activated CD4^+^ T cells, namely Th0 cells), A1 (Th2-like cells), and A2, in both ALV-J infected and control samples, demonstrated a potential differentiation correlation (Figure 5A). Cluster A0 (Th0 cells) is mainly located at the early stage of the pseudo-time trajectories, whereas clusters A2 and A1 (Th2-like cells) are mainly located at the late stage (Figures 5A,B). These results suggest that clusters A2 and A1 are likely differentiated from A0 and imply that cluster A2 might represent Th1-like cells. Interestingly, cells in the control PBMCs are mainly distributed in the A0 (Th0) and A1 (Th2-like) cell populations (Figures 5C–E). Conversely, ALV-J-infected PBMCs are highly enriched in the terminally differentiated A2 cell populations (Th1-like; Figures 5C–E), which indicates that ALV-J infection induced CD4^+^ T cell activation and differentiation into the Th1 phenotype. Finally, the branch-dependent differential gene hierarchy clustering heat map is shown in Additional file 9 in Supplementary Material; it contains the top 10 branching DEGs displayed in Figure 5F. Of note, *BRT-1*, *CSTA*, *ENSGALG00000046729*, *HPSE*, *IFI6*, *ITGB3*, and *TUBB1* may be associated with Th1 cell differentiation (Figures 5D–F).

### Cluster A2 (Th1-Like) Is the Vital Cell Type in Response to Avian Leukosis Virus Subgroup J Infection

**FIGURE 6:**
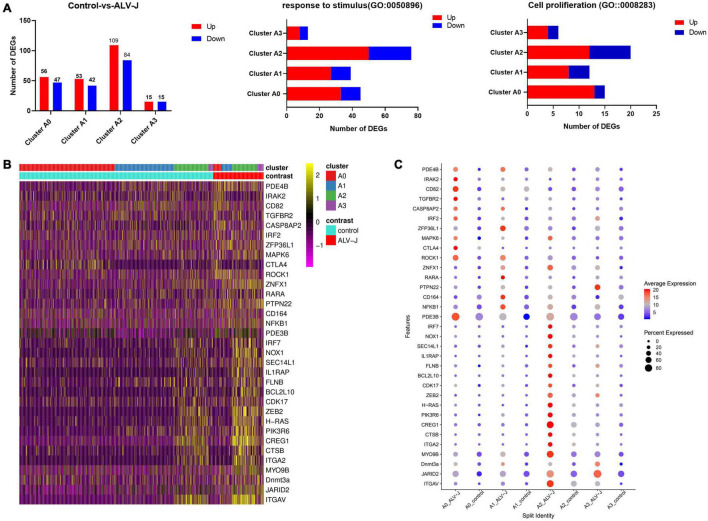
Landscape of immune-related gene expression in clusters A0–A3 of the ALV-J infected and control samples. **(A)** Histogram shows all the up-regulated (red) and down-regulated (green) DEGs, and the number of DEGs involved in GO terms “response to stimulus” and “cell proliferation” in cells of clusters A0–A3 from ALV-J infected chicken compared to those from the control. **(B)** Heatmap shows the normalized expression (*Z*-score) of all immune-related DEGs in cells of clusters A0–A3 from the ALV-J infected sample compared with the control one. **(C)** Dot plot representing DEGs expressed in clusters A0–A3 from the ALV-J infected sample is compared with those from the control sample. The intensity represents the expression level, while the size of the dots represents the proportion of cells expressing each gene.

Our above results showed that cells in clusters 6 and 7 might have played a potentially important role in the antiviral response during ALV-J infection based on their largely immune-related DEG expression (Figure 3A, Additional file 6 in Supplementary Material). Meanwhile, the T cell populations in clusters 6 and 7 could be further grouped into four distinct sub clusters (Figure 4). To further investigate each T cell population, we first calculated the DEGs between corresponding T cell population in the PBMCs from ALV-J infected and control chickens at 21 DPI using Seurat. Strikingly, the highest numbers of total DEGs and the DEGs enriched in the GO terms “response to stimulus” and “cell proliferation” were predominant in cluster A2 (Th1-like T cells; Figure 6A, Additional file 10 in Supplementary Material), suggesting that cluster A2 represents likely the most vital effectors among PBMCs of the ALV-J infected host.

Next, the specific DEGs of the four sub clusters enrichment in “response to stimulus” (GO:0050896) were exhibited using a volcano plot (Additional file 11 in Supplementary Material). Moreover, a total of 33 important immune-related genes were screened from clusters A0 to A3 and their expression levels were quantified in a heat map and a dot plot (Figures 6B,C, Additional file 10 in Supplementary Material). We also observed that most of these immune-related genes were highly expressed in cluster A2. It is worth noting that *BCL2L10*, *H-RAS*, *IRF7*, *NOX1*, *SEC14L1*, *IL1RAP*, *FLNB*, *CDK17*, *ZEB2*, *PIK3R6*, *CREG1*, *CTSB*, and *ITGA2* were up-regulated in cluster A2, rather than in the other three clusters (Figure 6C, Additional file 10 in Supplementary Material). Moreover, it is reported that H-RAS act as critical controllers of Th1 responses via transmitting TCR signals for the Th1 priming of CD4^+^ T cells (Iborra et al., 2011), which further supports the results we described above that cluster A2 may represent Th1 cells. Besides, we found that *PDE3B*, *RARA*, and *CD164* were only up-regulated in cluster A1, while *IRAK2*, *CD82*, *IRF2*, *MAPK6*, *TGFBR2*, and *CTLA4* were only up-regulated in cluster A0. Furthermore, the apoptosis-associated gene, *CASP8AP2* (Wang et al., 2018), exhibited increased expression in clusters A0 and A1, which could potentially explain their proportional decrease in PBMCs after infection. Taken together, these up-regulated genes could potentially serve as marker genes for chicken Th1-like cells (cluster A2), Th2-like cells (cluster A1), and Th0 cells (cluster A0), respectively.

**FIGURE 7:**
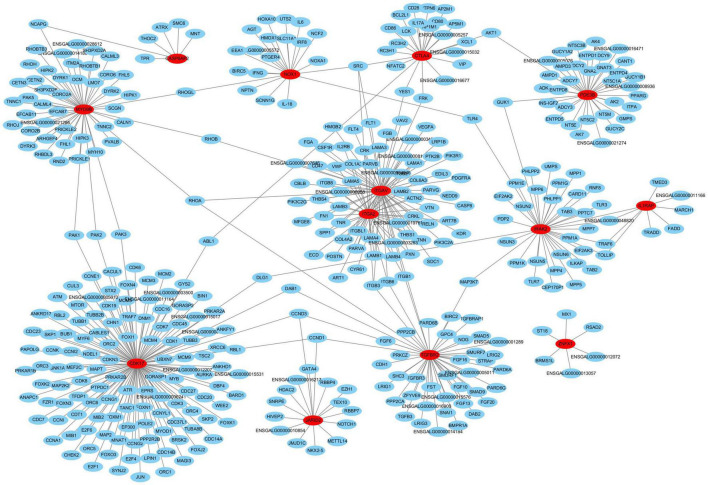
The interaction network analysis of the 33 candidate DEGs based on the STRING database. In this network, red nodes represent hub genes, and lines represent potential associations.

Finally, we conducted an interaction network analysis of the 33 candidate DEGs based on the STRING database (Figure 7). The results implied that the 13 DEGs marked in red are likely the more important hub genes. Specifically, four hub genes including *ITGA2*, *IL1RAP*, *NOX1*, and *CDK17* were up-regulated in cluster A2 (Th1-like cells, Figure 6C, Additional file 10 in Supplementary Material). Three hub genes including *IRAK2*, *CTLA4*, and *TGFBR2* were up-regulated in cluster A0 (Th0 cells, Figure 6C, Additional file 10 in Supplementary Material). Two hub genes including *MYO9B* and *ZNFX1* were up-regulated in Th1-like cells and cluster A1 (Th2-like cells, Figure 6C, Additional file 10 in Supplementary Material). Besides, identified hub gene *PDE3B* was up-regulated in Th2 like cell; *ITGAV* was up-regulated in Th1-like, Th2-like, and cytotoxic CD8^+^ T cell populations; *CASP8AP2* was up-regulated in Th0 and Th2-like cells; *JARID2* was up-regulated in Th0, Th1-like, and cytotoxic CD8^+^ T cell populations (Figure 6C, Additional file 10 in Supplementary Material).

## Discussion

Adaptive immunity, including T cell and B cell response, is the foundation upon which vaccines are developed. Despite decades of research, we still have limited insights into the ability of the avian immune response to pathogens at both cellular and molecular levels. For example, antibody level and T cell proportional change in the peripheral blood are usually used to evaluate a virus-induce immune response (Feng and Zhang, 2016; Ruan et al., 2020; Dai et al., 2020a,^2021^). Flowcytometry-based phenotyping and functional evaluation of chicken T cells are limited by reagents and methods availability (Dai et al., 2019). Fortunately, advances in scRNA-seq allow us to at least partially overcome these defects to gain insight into the molecular signature of avian immune responses to viral infections. Previously, we found that ALV-J viremia was eliminated by 21 DPI when an up-regulated CD8^+^ T cell proportion and low antibody levels were detected (Dai et al., 2020a). In the current study, we used 10x scRNAseq on PBMCs in ALV-J infected chicken and uninfected chicken (control) at 21 DPI to characterize two major aspects of immune response against the invading pathogen: the immune cell composition and their responses to infection.

In this study, eight distinct cell populations in chicken PBMCs were identified following analysis of scRNA-seq data. Strikingly, we found that known T cell populations (clusters 6 and 7) and B cell populations (cluster 8) occupied a very small proportion in chicken PBMCs. Of note, ALV-J infection had an obvious impact on the cell composition of PBMCs. Specifically, the total number and proportion of T cells, B cells, and the cells in cluster 2 were distinctly reduced in PBMCs from ALV-J infected chicken compared to those in the control PBMCs. Furthermore, up-regulated *ITPR2* (van Es et al., 2007) and *JARID2* (Gennart et al., 2015) expression might be involved in T cell apoptosis in PBMCs from ALV-J infected chicken (Figures 1C, 3B, Additional file 6 in Supplementary Material). On the other hand, the proportion of clusters 0, 1, and 3 were obviously increased in PBMCs from ALV-J infected chicken compared to that of the control PBMCs, which could be associated with the up-regulation of the anti-apoptotic gene, *BCL2L10* (Zang et al., 2015). In future, experiment *in vitro* about ALV-J infection of cells of each cluster are needed in order to further confirm our above observation for exclusion scRNA-seq technical or sampling effects.

Additionally, Pearson’s correlation analysis showed that clusters 0, 1, 2, 3, 4, and 5 exhibited strong correlation, but much less so with the T cell and B cell populations (clusters 6, 7, and 8; Figure 3C). Unfortunately, clusters 0, 1, 2, 3, 4, and 5 were unable to be defined based on the limited known chicken cell-type markers and the five most highly expressed genes in these clusters. Interestingly, the top expressed genes in cluster 4 were mainly chicken ISGs including *RSAD2*, *TRIM25*, *OASL*, and *IFIT5* (Dai et al., 2020b), which implied that cluster 4 contained important antiviral immune cells (Figure 2C). Moreover, *IRF7* which is involved in IFN-β signaling and ISGs inducer (Cheng et al., 2019; Dai et al., 2020b), revealed up-regulation in clusters 0, 1, 2, and 3 response to ALV-J infection at 21 DPI (Figure 3B, Additional file 6 in Supplementary Material). According to these results, the function of clusters 0, 1, 2, 3, 4, and 5 may be related to innate immune response at the early infection stage. The specific cell types and function of these clusters need to be further confirmed in future studies. Besides, the inability to detect the DC or NK cell population and so on may be largely due to limited cell numbers for scRNA-seq in this study. In future, more samples and cell numbers were necessary for identifying more cell types that occupied very small proportions in PBMCs.

Most DEGs were detected in the T cell population (clusters 6 and 7) response to ALV-J infection at 21 DPI (Figure 3A, Additional file 6 in Supplementary Material). Furthermore, the T cell population could be further divided into four distinct cell populations including CD4^+^ T cells (Cluster A0), Th2-like cells (Cluster A1), Th1-like cells (Cluster A2), and cytotoxic CD8^+^ T cells (Cluster A3), based on their marker genes expression and pseudotime analysis results. Here, pseudotime analysis implied that CD4^+^ T cells in chicken PBMCs could become terminally polarized to a Th1 or Th2 phenotype. Moreover, ALV-J infection induced CD4^+^ T cell activation and differentiation into a Th1 phenotype was likely associated with the expression of the top 10 branching DEGs (Figures 5D–F). In addition, the Th1-like cell population (Cluster A2) was vital in response to ALV-J infection at 21 DPI based on the expression of largely immune-related DEGs. Compared to the control PBMCs, the proportion of Th1-like cells (cluster A2) and cytotoxic CD8^+^ T cells (cluster A3) were increased in the T cell population of PBMCs from ALV-J infected chicken. It is also known that Th1 cells can help cytotoxic CD8^+^ T cell activation, survival, and memory generation (Huang et al., 2007). Conversely, B cell proportion was decreased, and fewer DEGs were detected after ALV-J infection (Figures 1C, 3A). It is also reported that ALV-J infection inhibits the proliferation, maturity, and responing of B cells (He et al., 2019). Therefore, we speculated that it was the T cell response, including Th1-like cells and cytotoxic CD8^+^ T cells, instead of the B cell response that eliminated ALV-J viremia before 21 DPI. Our previous animal experiments also verified that T cell response as opposed to humoral immunity was the key factor defending against ALV-J infection (Dai et al., 2020a).

More importantly, we identified 13 hub genes for the first time that were up-regulated in each chicken T cell population after ALV-J infection. Of note, *CDK17*, reported to inhibit porcine reproductive and respiratory syndrome virus (PRRSV) infection (Bai et al., 2019); *Nox1*, reported to suppress influenza A virus induced lung inflammation and oxidative stress (Selemidis et al., 2013); and *IL1RAP*, reported to negatively regulate Transmissible gastroenteritis virus (TGEV) induced mitochondrial damage (Zhao et al., 2018), were all up-regulated in the Th1-like cells of PBMCs from ALV-J infected chicken. The information reminded us that *IL1RAP*, *NOX1*, and *CDK17* could potentially serve as marker genes of Th1-like cells exerting antiviral function. Furthermore, we found that *ZNFX1* was up-regulated in Th1-like and Th2-like cells, and was involved in inducing IFN gene and ISG expression (Wang Y. et al., 2019), which indicated the complexity of antiviral immunity in T cells. On the other hand, *IRAK2*, reported to potentially suppress avian infectious bronchitis virus (IBV) infection (Liu et al., 2018), exhibited up-regulated expression in activated CD4^+^ T cells (cluster A0, Th0 cell) of PBMCs from ALV-J infected chicken. Interestingly, *CTLA4*, a critical co-receptor for Treg cell function (Friedline et al., 2009; Kerdiles et al., 2010), and *TGFBR2*, the important TGF-β receptor suppressing proliferation and terminal differentiation of antiviral CD4^+^ T cells (Lewis et al., 2016), also exhibited up-regulated expression in activated CD4^+^ T cells. In the current study, we did not identify the two subpopulations of chicken regulatory T cells (Treg cells) including TGF-beta^+^CD4^+^ T cells and CD4^+^CD25^+^ T cells (Shanmugasundaram and Selvaraj, 2011; Gurung et al., 2017). We hypothesize that Th0 cells may negatively regulate T cell response to homeostatic control through *CTLA4* and *TGFBR2*. Therefore, further scRNA-seq experiments with additional pathogens and preferentially at various time-points during the infections are needed in order to further confirm our above observation. Ultimately, such function should be demonstrated in a functional assessment using isolated cell types. Besides, given that the annotation of chicken genome, GRCg6a is incomplete, the information and function of many novel genes are not clear, which limits the effective analysis of scRNAseq results.

In summary, our scRNA-seq study based on PBMCs from chickens generated a rich data resource that could be mined in future experiments to address the function of these cells throughout development and in response to pathogen infection. The “marker genes” that were assigned to different clusters need to be verified using specific antibodies to their gene products. To the best of our knowledge, the mark genes for each T cell population involved in ALV-J infection are identified here for the first time. Moreover, using pseudotime analysis, we found that chicken CD4^+^ T cells could differentiate into Th1-like and Th2-like cells. With respect to the control PBMCs, ALV-J infection had an obvious impact on PBMCs composition. B cells were decreased and inconspicuous in response in PBMCs from ALV-J infected chicken at 21 DPI. Cytotoxic Th1-like cells and CD8^+^ T cells are potential key effectors in the defense against ALV-J infection.

## Data Availability Statement

The datasets presented in this study can be found in online repositories. The name of the repository and accession number can be found below: SRA, NCBI; PRJNA687808.

## Ethics Statement

The animal research projects were sanctioned by the South China Agriculture University Institutional Animal Care and Use Committee (identification code: 2019076, 10 June, 2019).

## Author Contributions

XQ, XL, and ZL assisted with data analysis. MD designed the study, performed experiments, collected and analyzed data, and drafted the manuscript. ML coordinated the study and revised the manuscript. All authors have read and approved the final manuscript.

## Conflict of Interest

The authors declare that the research was conducted in the absence of any commercial or financial relationships that could be construed as a potential conflict of interest.

## Publisher’s Note

All claims expressed in this article are solely those of the authors and do not necessarily represent those of their affiliated organizations, or those of the publisher, the editors and the reviewers. Any product that may be evaluated in this article, or claim that may be made by its manufacturer, is not guaranteed or endorsed by the publisher.

## Footnotes

1
https://support.10xgenomics.com/single-cell-gene-expression/software/overview/welcome

2
https://github.com/alexdobin/STAR
