# Supplementary material for: Chicken Peripheral Blood Mononuclear Cells Response to Avian Leukosis Virus Subgroup J Infection Assessed by Single-Cell RNA Sequencing
Source: Front Microbiol. 2022 Mar 14;13:800618. doi: 10.3389/fmicb.2022.800618 (PMC8964181; doi:10.3389/fmicb.2022.800618)
Supplement: Supplementary file 1 [file Table_1.DOCX]

Additional File 1. scRNA-seq data statistics

| **Sample** | **Number of Reads** | **Valid Barcodes** | **Number of Cells (before_filter)** | **Number of Cells (after_filter)** | **Median UMI Counts per Cell** | **Median genes per Cell** | **Mapped to Genome** |
| --- | --- | --- | --- | --- | --- | --- | --- |
| **ALV-J** | 388,383,450 | 95.4% | 20554 | 13767 | 503 | 337 | 84.10% |
| **Control** | 400,871,000 | 96.3% | 13126 | 9797 | 450 | 312 | 83.50% |
