# Supplementary figures and images for: Chicken Peripheral Blood Mononuclear Cells Response to Avian Leukosis Virus Subgroup J Infection Assessed by Single-Cell RNA Sequencing
Source: Front Microbiol. 2022 Mar 14;13:800618. doi: 10.3389/fmicb.2022.800618 (PMC8964181; doi:10.3389/fmicb.2022.800618)

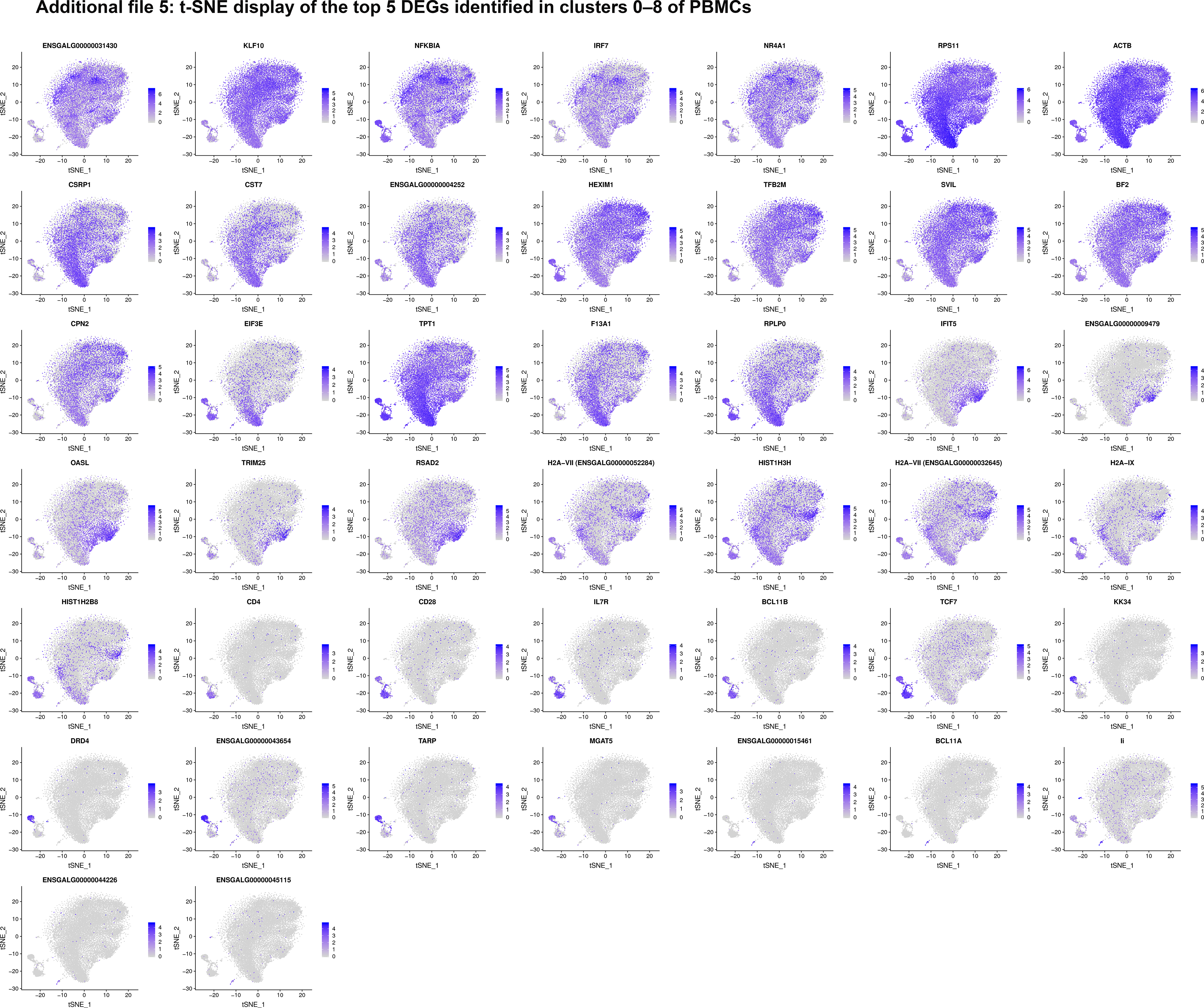

Supplement: Supplementary file 8 [file Image_1.TIF]

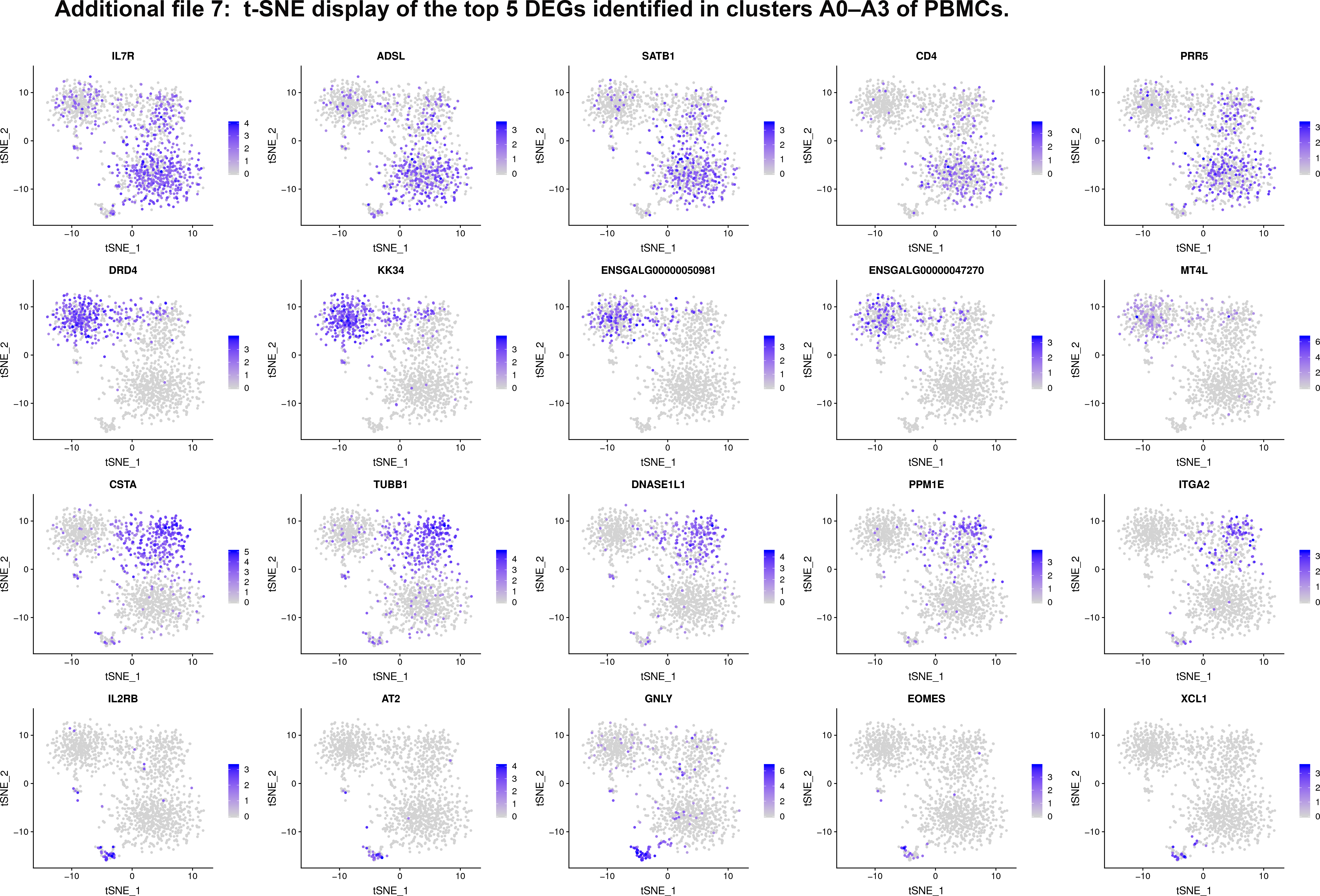

Supplement: Supplementary file 9 [file Image_2.TIF]

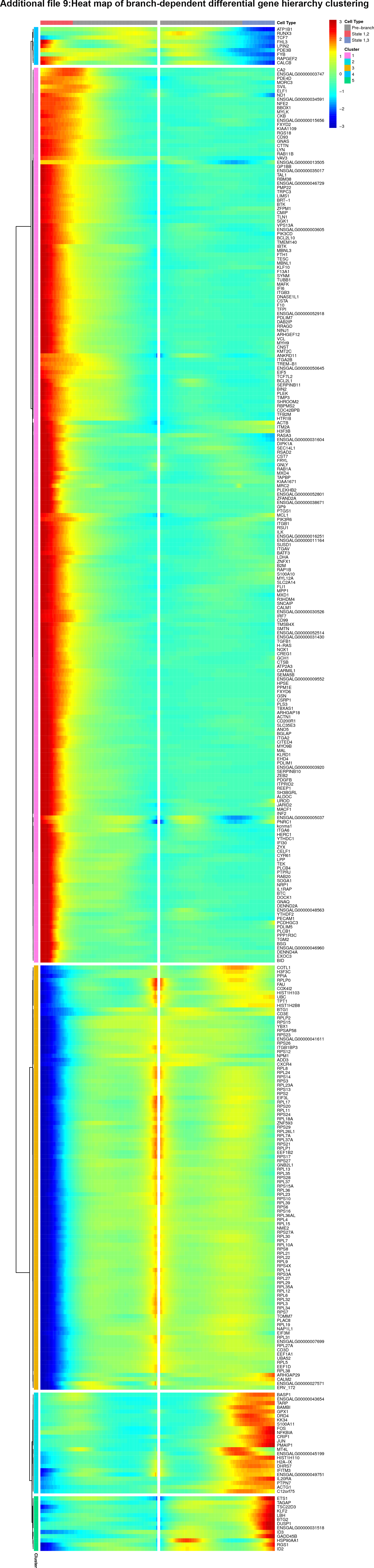

Supplement: Supplementary file 10 [file Image_3.TIF]

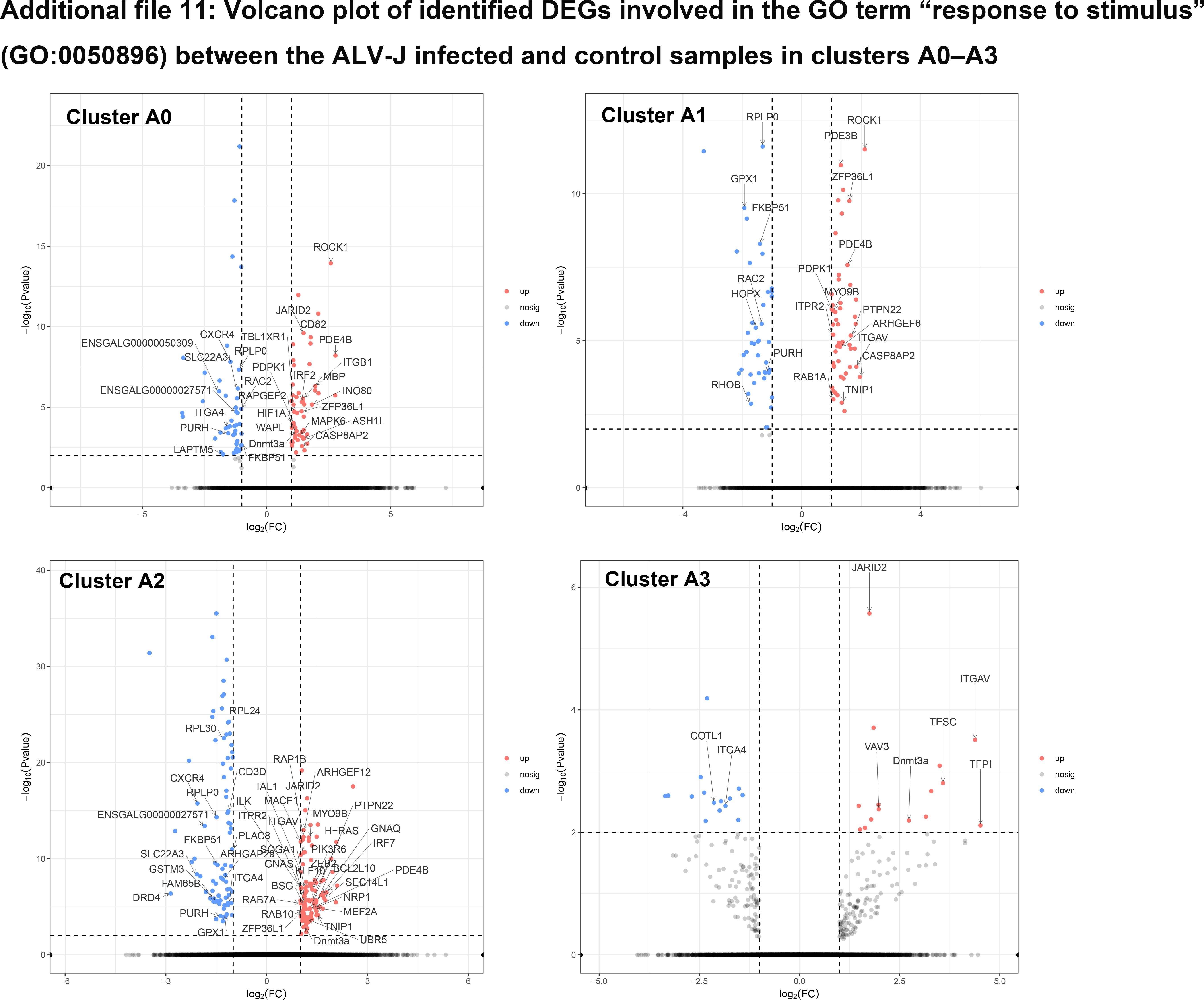

Supplement: Supplementary file 11 [file Image_4.TIF]
